# Supplementary material for: Functional Characterization of the Thrombospondin-Related Paralogous Proteins Rhoptry Discharge Factors 1 and 2 Unveils Phenotypic Plasticity in Toxoplasma gondii Rhoptry Exocytosis
Source: Front Microbiol. 2022 Jun 9;13:899243. doi: 10.3389/fmicb.2022.899243 (PMC9218915; doi:10.3389/fmicb.2022.899243)
Supplement: Supplementary file 1 [file Data_Sheet_1.zip › Supplementary Data Sheet 1/Supplementary Figure 1.DOCX]

MIC15 MVFRATREPFRLPLVAAFIALFLLKGVTCQLQLGTSTLQKNMWTTVKFDEPMIDPVVFVSPPEAPTNSFALVLIGGVTTSGFRARVYFPSCEYSPSGGTLYAVRWLAVEATSAGYYDSSN 120

MIC14 MGARAVWSAGS--------DAGRT-----------------------WLLRAGPVCALLVLSLCSFLFLV 39

MIC15 RQTIDWTAHAVTLASSGTIPAGYVWSQSFSAWEDRPGTPGVLIGVQNHREMISAMYWSSEYLNPLVINSNSGGTLTFSFLRQISHRIGFTLKVGIFQYDARRGAVINGVNLVAQRYDLKV 240

MIC14 CK----SSAEARATTPVS------EPREATTVRENSETKPLSQGQNAAA----NPARRS-VSTSQALSFFDSMRPGPYKGRSSVYSNAGPD**C**YNLAATTLTVPKGL**C**TET**C**VLVWTDGKK 144

MIC15 NEVLPVADVVAGGTTPATFAVQWVTPTEAPL-ALVVKPKLLPDGSYSESLVAVEDCRNGATYEPHAFDVTHAFTGN--PASTAHRINGGPN**C**FRPSASVPTWLTEP**C**KLA**C**ESLWTANSD 357

MIC14 QYAS**C**KDRVT**C**FLRDYTSRQVQAAQAGIPRKLSAAISRE**C**SFVSKSPHLPLDQFQHMFILSASDVEHHGSWDAASA**C**TAGQR-LDVAGALVGPKAVVAGTAAESVG**C**ESINVTSHLKDI**C** 263

MIC15 DYWN**C**DDVNA**C**FETDFRSAETLMALPQIDESIADVLVTS**C**NFATRTADL-IKQYEYAVNLKVDDIAIGTMWSTSSH**C**GGDKMEVEIVGAFYEKLGVS---ILTGST**C**EVQDVSREVEAL**C** 473

MIC14 AEAIKNPTER**C**IIEPSVYSNIISKF--S**C**NVKETPYLALYVT**C**IDVKVDDDVG**C**KVVTPASSQPDPEGG**C**T**C**PSGTVQ**C**SLDESRLYGDVMSGIA--YGYTVHTKHNRGFKNGNVTA--- 376

MIC15 KAE---GGIG**C**SISAAQIYELIMNYPGV**C**FALEDAELQIFFN**C**TFA-STSPFD**C**ELYE-SKGFDQENGD**C**M**C**PNALAA**C**TREEATFKSGWLQNLNKSYQYMLHGQIRYVAKSSVFQAFYE 588

MIC14 -------FQTPF**C**SGATVKVA**C**KNAPDSASKKGEPATTAWLDRGPN**C**YKPTDSDNRQM**C**KFA**C**LKLWNPADGALVN-----VNTPFDDFKDRIKSGRLTVMELTADGNAAIRS**C**DWAKRI 484

MIC15 APETSSAGDNTL**C**ARMNTIVV**C**KN--------PTPVSAVVGNRGPN**C**YVIEH--GSQY**C**HLP**C**LTAWDAFLAAKDLPQQSDYQAEFERFWLSFDFPAIP-PHTSQSLLEQLQR**C**RFATRP 697

MIC14 RFAPAEQYEYETWTTSEA-----LSNF**C**PNSGTLQIVRAFLGCSYSLVDGNESQN**C**NYVDITKEVTKK**C**GSAWRSSKKG**C**TLGADELVAEADA**C**ETSCEGEWGIHLYYN**C**TPET--P-SA 596

MIC15 ATAPLQQYEDEIDVQLPDYGAVIIPLG**C**DKLHHREVVQALVGSASSIMLGVHEPG**C**RYEDATETVQRE**C**KAAVSARKTT**C**EIDSSLFTSKDIL**C**NTSTIK---LIIRGT**C**IPGPYLPTNY 814

MIC14 S**C**SLHQATTGLDETENEYA**C**G**C**PYLAEM**C**DLEQAE-TSTVWKDTVRGYTVAILKGNLTYGLNGGYKRYHTTTV--SSDT**C**ES-TGSRVL**C**KPPPASIELSPPNQTPD**C**TTSFSTKTGASS 712

MIC15 T**C**ALYP-TTGVV-SRDGRS**C**T**C**PNSAYP**C**TYEEGQLTASRWKNEVNTGATVSLANNVVWTAPPGTSEYTYTHTDAYDYV**C**STDEHHFVL**C**KDLT-PSALPLTEREPH**C**LNSFSVKPDAST 931

MIC14 SDDGL**C**VNECIIATQGE**C**SKATNAWL**C**VVKKVSN**C**FIPNKAKST**C**FIETEVGNYQAEFQS**C**S**C**PEASMP**C**TEEEVEATRYEWEPTFAPDNAYI--VVAPN-KVLG--PTKTIQPDYGMAA

MIC15 VDDRD**C**QDQCASLFATR**C**RQSFYKWL**C**VAQGLPE**C**YIPNTDSFT**C**QIDTNPSAYNYSYGS**C**G**C**SGAYPP**C**SRNEANATRLDWITAFRALSHKKGVVVARGKQALWTENPDRWRYENGMLQ 1051

MIC14 EPG**C**GS-SRYKRVF**C**RGTSGNTSVTRYDATAD**C**ENARSLDASVISDFE**C**RYGCSTRIKK**C**KAIMTQNPD--VHESEYA**C**YTAQLNTIPKLRQ**C**LVESKLVDPETGVGSMEAGFIVVTQEW 827

MIC15 GGG**C**LNNSWFVGVF**C**GAHLSVTPPARGDVLPL**C**SSAAKVENPDTPYPA**C**RVLCAQVLNE**C**KKVFSTTEGAAAYASVFD**C**FKARGK-GTTLDN**C**TYELGPENRATGVAELHTGWTVASASW 1170

MIC14 TSVAFKKEIENPV-VVTSLPATTNAVPVIQVKGVNSTGFKIRMKNDF**C**SVGF-ASPYTSVGWLASSQGTFLVSGAKSYVRVGTTLASL-------------NQPTTIRHLPRMNSAAPLV 1049

MIC15 SRVLFDVEFADPPVVFLGIPKDTQPFYTPSVRLVTKTSFEVKLYRNN**C**GLDDTRSSSAPVSWMAIPEGAYLTDFVENPVRVMKLPMTTRTPTVLTFSLSGLKEPEEMVALAQVQDVTPTS 1290

MIC14 FLQHEDSEGGSGNYIVASTVTASSASEATFKLTLVGS-GQPSEPIVIGYMIFDEIRQEYC-SLGCQLNGISMQTDYADSNERWTAESTDNVILAGQTVVYGSVVDPDGTSPVFVSKHDVA 1167

MIC15 VST-------DRVAVVIS-----NLKTNSLELSLVVDESVNFSTVAVGILISGKQNPDLAAGLSPLLNRYHLQT------FRIPAAAYSATSILGEGLYLS-----GGIMPHVFAAAIQT 1387

MIC14 WSSGNEIRSEGGV--------------ILDWRYAVPGGKDEHARSH-----------------PSGAQTSDSKTSKNDIGESLMQVSENLSVPGARSGWYPAIDIIYNSK**C**TVGSVSRTN 1256

MIC15 RSMTKELNESDRVVISRRATTTPFTWSALLWKKTCEAKHMFEAVENPSDLIIAGIYVEARKDEPSAILGNRHDLCLSFIGQSFESGVVATCMGACRSALPASVQLHCQDE**C**AL------N 1501

MIC14 IFPAAGGRKNSVSRRLVALLYVSSGTSEISSSSFPPYGAD**C**ASAAGKKNYAAAE**C**AAQ**C**EAT------------VEGCTDS--DEDAWP**C**FFRQFPIDQKKK**C**YITAVAS------DLAA 1356

MIC15 IFAPSVH---------------------------TKCMTD**C**ESLL---VAQYEE**C**AGQ**C**TATGTASCQRKCKQFLGNKCDSTASSDVAA**C**LEFVTPPSVFEQ**C**TLLVEYSVSEGGPSDGF 1591

MIC14 TSTTTAPPVDKDER**C**VLVDMSEAAYSAERGWDPETMT**C**K**C**PNDAPA**C**SAEQATYNLINHSSVALGQGAICAAATGTVSGIARHFKRSTLDL**C**GD--LSKLSPLSWEQLPLPPYMADDVIL 1474

MIC15 EIEEETTPSQTYEN**C**IIVDMRDERFTAFTGWDSRVAS**C**K**C**PNDFRA**C**TSETVNTQNHWRTELLASAGLCKEQPDGRFNAVTQQLWSTTGDL**C**ALAADEQQPPISWASNMLPAYVDQDIVL 1711

MIC14 DDEVIRSGHYL**C**TKSYHHVF**C**PSSLITTSTTTPEPKWDWPARQDCVPGEWTSWSK**C**SMD**C**YVGSGALPVKSRSRSVLVDRRGTGNVCVLEDHTLCKVGSEVPY**C**EDL**C**WITDWGDWSG**C**E 1594

MIC15 DNSTIRSGDYQ**C**REIWHYVV**C**PAAATSTTTQEPPVPPFTATLNTAIVGEWGEWSA**C**TGT**C**FSQWW-TPKRTRTRLVLAELSHSQ-IPSVSE---TATCLDLPP**C**GTV**C**WEREWTEWSE**C**K 1826

MIC14 QKVLQIGQAAVRARTKLRHIFMGTGGV**C**SSDH-IQYDYSGCK------------------------------------------------------------------------------ 1635

MIC15 LFTIVMGQGLEYFRQQIKPVFDFVEEA**C**GLDEHERYERCGEEQDNGETPATSTLQTDSLLETRSLTVSHGTAARALPRVSMDPALERPSFASLHGTRSHVPASTDAKIIERRKGIMSRRR 1946

MIC14 -----------------------------------------------------------------------------------SGMDSSGGREDDSAHSM------------------VE 1654

MIC15 SVPPSGYLEETAEQVAHGGESEQSGKASQNGSRRHRASRKQKRDLESIYSDASVRGSGESTLHGTGTNAYRDQIEWTSKSSSRSAIRDSGARGAEAGTSLLQKARRARRGAGRFRKSRSQ 2066

MIC14 QKSRVTDAG**C**KKSSGWSA**C**SLP**C**QQPGANAVTQQFQLGLPETVTASTTSY**C**ASRS---RACVEKKPP**C**VLDVAPD**C**SLVKPRYDSPEEALY**C**HEL**C**NNALTR**C**REQAALQFQT----QWE 1767

MIC15 ARNQTPDKS**C**SVVSEWSG**C**DSP**C**LPHKG-STPRRYRLAVPGQNEA---NY**C**TVPLSGTEHLCTDLPQ**C**A-HPNFD**C**AKVTASRLTDEDIAA**C**KAV**C**VEVVKT**C**ETMMSAVFSLYTSLEQC 2181

MIC14 CIMAFINSSGVTGR**C**QIQKGAVSKTTLRPPV**C**FLSRTRQVDASKSLSFLTTDK---------------------------------------------TSGRGS**C**M**C**AVTNSVP**C**SPEEV 1842

MIC15 ALVQFQEYEQFPGQ**C**HIPEEY--TSTRRPTK**C**FPSRTRRRVASGELPFIFQVSTASGTSPSATSDAASSVAESFVSTGTASAPSSRVMNALAFEASASQTSIDS**C**E**C**YDPADEP**C**TAQEA 2299

MIC14 ALS-FDDWAKQLGSTV**C**PF--GQLGAFFNSPNSKGSADSFVYFGASDLGRVH**C**PISKSS-------DLGSRFPTFTEFESPESMNNF**C**ENGLPFWQEKREPLVD-------**C**RQVIPKN- 1944

MIC15 RDSLFDSLYLFLTHPL**C**AESPAAEGALFTMSEPNQTADAASYFALRGLGRMH**C**PVPMYKSSTEVKGVLTPKRVTFSEFKTKEDLNEF**C**HKGLSNWRQNVPPEIIGSASFPN**C**MLVTPREG 2419

MIC14 ASTTD**C**PGR**C**HKAVVN**C**MV---SNDSLDN**C**INEALSAGDFAEN**C**ELQSSVKLGKGLMF**C**KRIRTD**C**EYSEWSEWSE**C**SRT**C**RSGGDDEESVRVRGRKLLVAAEHG-GS**C**DADIEDSHGGS 2060

MIC15 AEPQD**C**ALL**C**SETMSS**C**SAASLSFVELSQ**C**VQDKLTESDFYSK**C**SAPEVLAPGEGIIL**C**KKKVST**C**DYTEWSEWST**C**TAT**C**FNWDEGVIPLRVRSRDFASSSADSRVL**C**RLE----SQND 2535

MIC14 LSDVQL**C**DWLPP**C**DALADTE----TYVIMPKPEPKLADWSATRTTTTTVIP---DLQNENKIL**C**TIVDMAD-FSKSHRGYDTETES**C**K**C**PYNARV**C**SRTEAVNSRDNWDELMQTV**C**ESNG 2172

MIC15 AIQTEK**C**DWMPV**C**PEAEGEEEDDATGGVEPRGEPIVPPWSPERPTDENNQAMGSEDIVSGTVE**C**YVTNMGTIMTSYYRGYNQEYHG**C**N**C**PGGRRP**C**TRAEAVASLDLWTKDSGGL**C**DQDM 2655

MIC14 QGQILAQGMETFS**C**SDRVFRSFRGTLGQTPEAH**C**KSEDATYIF**C**EDGAIDNDLIFTEVMMSIITGVVMGIAVVYWAIQYSGDVQKVLGLAGRYVELTNLLHEVSQEPQAHEEEKAEEGEG 2292

MIC15 ATMISAAEGEAFF**C**ATGSFGKIDTS---LSESS**C**ASSEYQYVL**C**EGHPYEGIANLTTWVICLLLGVGGGICFVLSCVQYSSDIQKLLGLAGSYPVLVQNVTELQERESHKL-RRQGNISA 2771

MIC14 PDEDAEPEAAVEGNSEFDCNPRAKEENGEESEQDASDSESGSDDDDDIVSEE 2344

MIC15 TSERSDAHALSLSDSGWDVDGNQS--AGSAFPEEE-PWQFEDRDEEPLLSTRKYSRNLGSAGIPEPSEIGQTSPTQQRVPRASLAAQRSTCQLSSRLGQANSPEQSLERRSLKLRDSHAD 2888

MIC15 VELQRTLRKEMKGNQVWDKTV 2909

**Supplementary Figure 1.** Sequence homology between MIC14 and MIC15. The full length amino acid sequences of the two proteins were aligned with Clustal Omega. Conserved amino acids are boxed in yellow, invariable cysteine residues are highlighted in red. The main amino acids domains or elements are indicated by horizontal bars according the following color code: TSP1 domain (red), H-lectin domain (grey), transmembrane region (blue), leader peptide (green), signal-anchor sequence (orange).

**Supplementary Figure 1**
