# Supplementary material for: Functional Characterization of the Thrombospondin-Related Paralogous Proteins Rhoptry Discharge Factors 1 and 2 Unveils Phenotypic Plasticity in Toxoplasma gondii Rhoptry Exocytosis
Source: Front Microbiol. 2022 Jun 9;13:899243. doi: 10.3389/fmicb.2022.899243 (PMC9218915; doi:10.3389/fmicb.2022.899243)
Supplement: Supplementary file 1 [file Data_Sheet_1.zip › Supplementary Data Sheet 1/Supplementary Figure 3 Caption.DOCX]

**Caption to Supplementary Figure 3**

**Supplementary Figure 3.** Endogenous tagging and localization of MIC15. **(A)** Schematic representation of three MIC15 variants in which the tags smMYC, Ty or HA were introduced at the N-terminus, upstream of the TMD or at the C-terminus, respectively. **(B)** Western blots showing recognition of the differently tagged MIC15 proteins by the relative anti-tag mAb and by the anti-MIC15 rabbit serum MIC15Nt. The dense granule protein GRA1 or the inner membrane complex protein GAP45 were used as loading controls. **(C)** Immunofluorescence localization of Ty- and HA-tagged MIC15 variants in intracellular tachyzoites stained with the corresponding anti-epitope mAb. For the localization of smMYC-tagged MIC15 see Figure 3D. Nuclei are stained with DAPI. Scale bars, 5 μm.
