# Supplementary figures and images for: Functional Characterization of the Thrombospondin-Related Paralogous Proteins Rhoptry Discharge Factors 1 and 2 Unveils Phenotypic Plasticity in Toxoplasma gondii Rhoptry Exocytosis
Source: Front Microbiol. 2022 Jun 9;13:899243. doi: 10.3389/fmicb.2022.899243 (PMC9218915; doi:10.3389/fmicb.2022.899243)

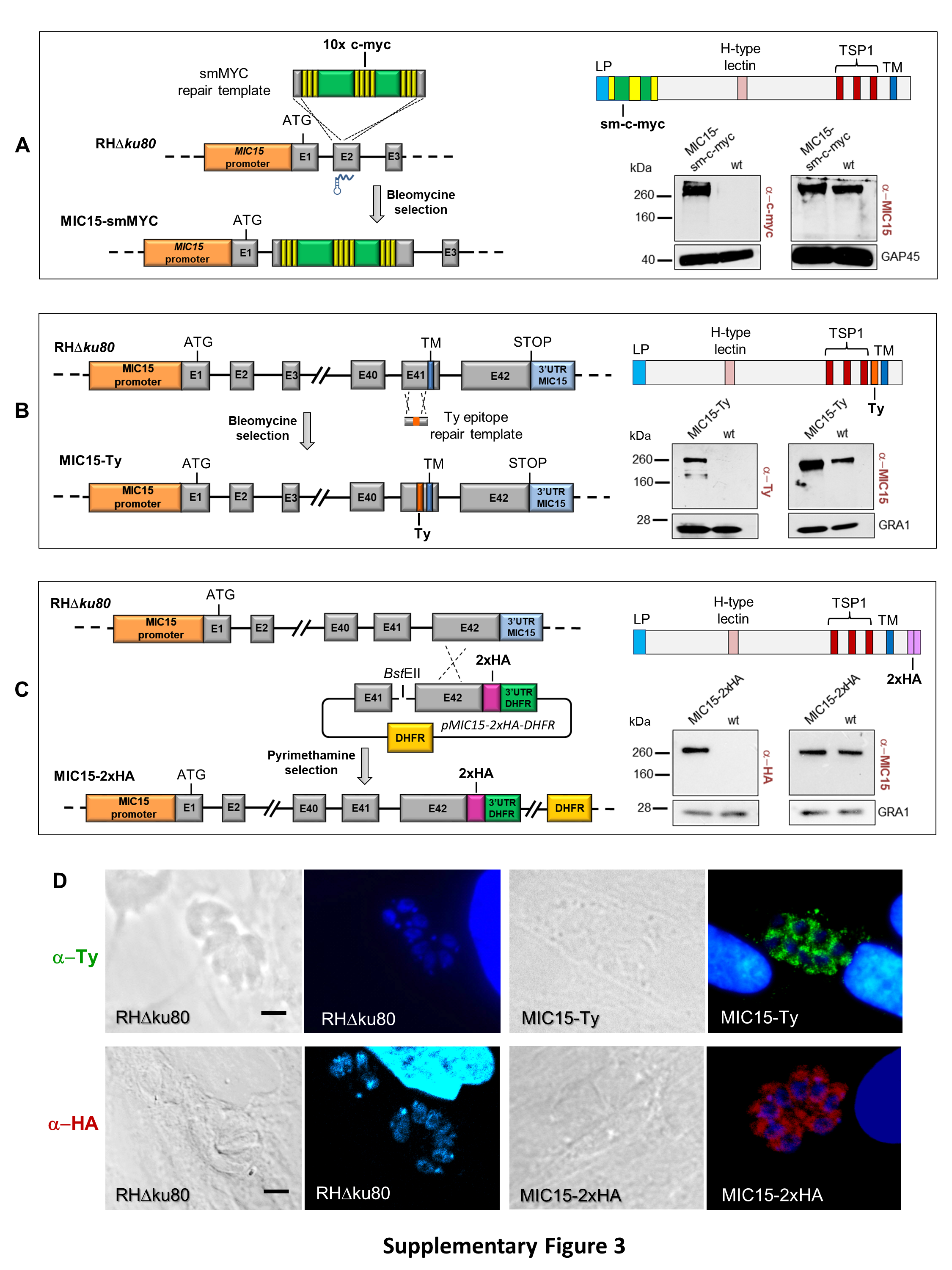

Supplement: Supplementary file 1 [file Data_Sheet_1.zip › Supplementary Data Sheet 1/Supplementary Figure 3.TIF]

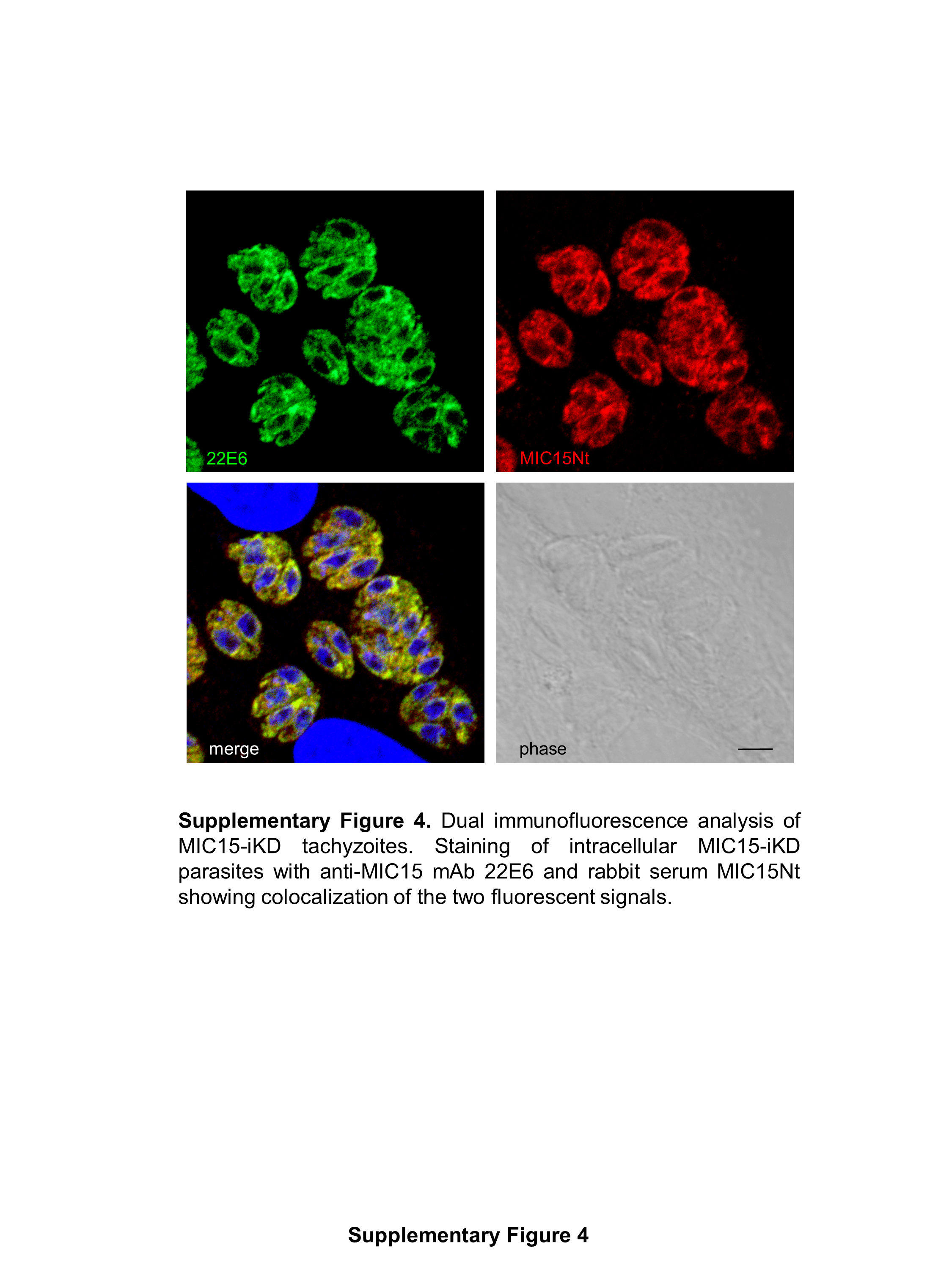

Supplement: Supplementary file 1 [file Data_Sheet_1.zip › Supplementary Data Sheet 1/Supplementary Figure 4.TIF]

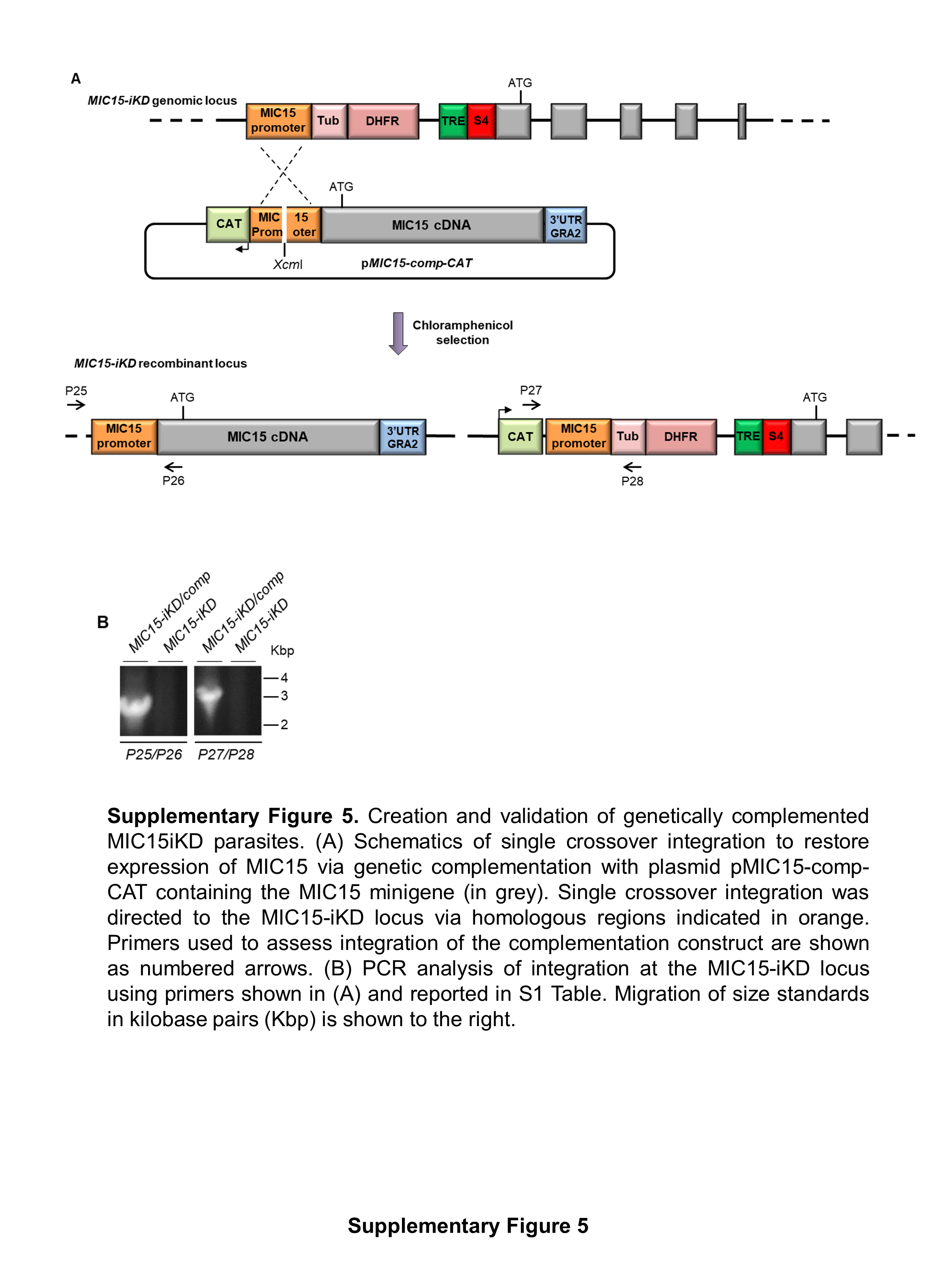

Supplement: Supplementary file 1 [file Data_Sheet_1.zip › Supplementary Data Sheet 1/Supplementary Figure 5.TIF]

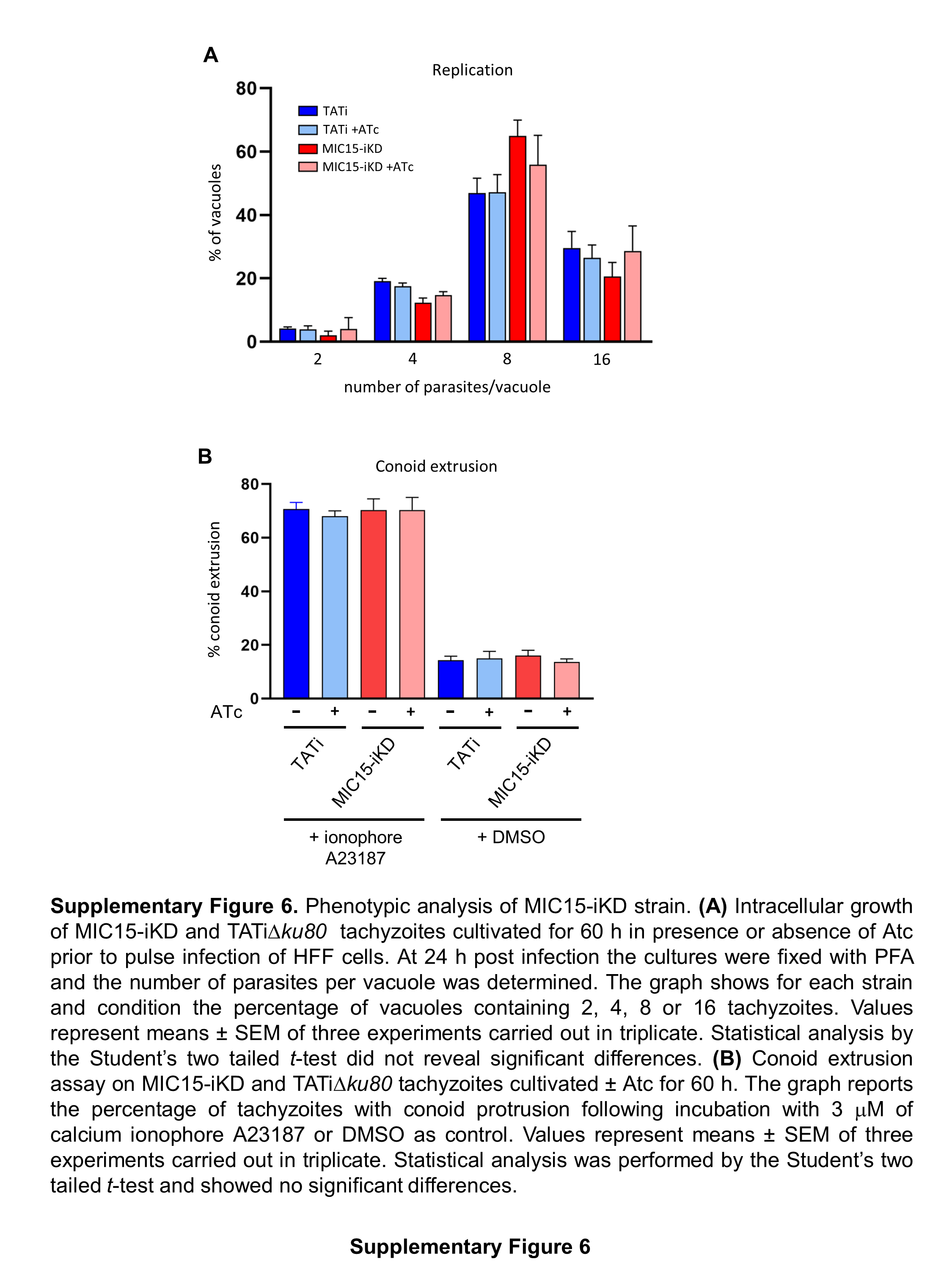

Supplement: Supplementary file 1 [file Data_Sheet_1.zip › Supplementary Data Sheet 1/Supplementary Figure 6.TIF]

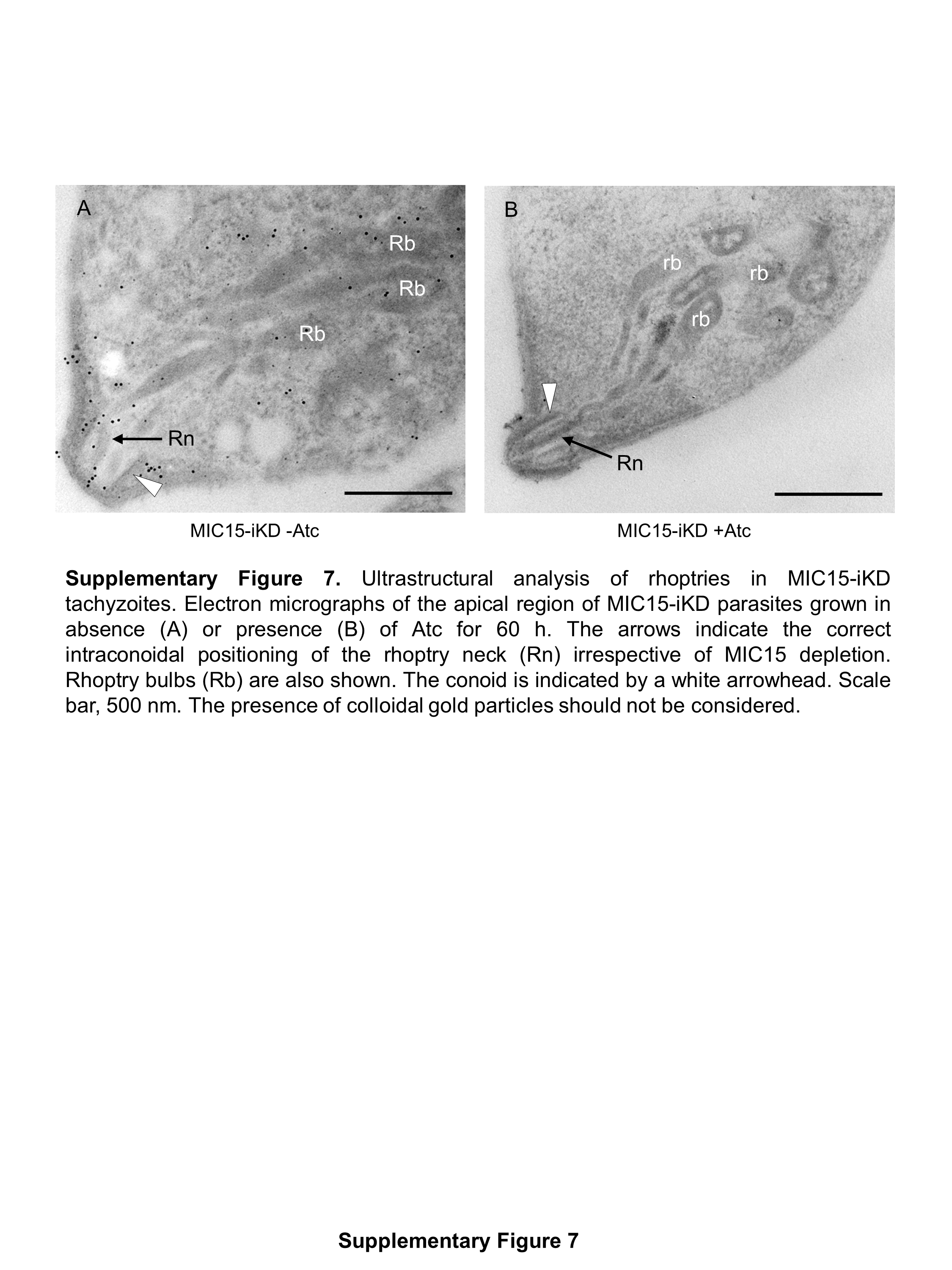

Supplement: Supplementary file 1 [file Data_Sheet_1.zip › Supplementary Data Sheet 1/Supplementary Figure 7.TIF]
